# Caring for women living with HIV: gaps in the evidence

**DOI:** 10.7448/IAS.16.1.18509

**Published:** 2013-10-01

**Authors:** Mona R Loutfy, Lorraine Sherr, Ulrike Sonnenberg-Schwan, Sharon L Walmsley, Margaret Johnson, Antonella d'Arminio Monforte

**Affiliations:** 1 Women's College Research Institute, Women's College Hospital, University of Toronto, Toronto, Ontario, Canada; 2 Royal Free University College Medical School, London, UK; 3 German AIDS Society, Munich, Germany; 4 Toronto General Hospital, Toronto, Canada; 5 Royal Free Hospital, London, UK; 6 San Paolo University Hospital, Milan, Italy

**Keywords:** gender, women, HIV, safety, pharmacokinetics, stigma

## Abstract

**Introduction:**

In the management of HIV, women and men generally undergo the same treatment pathway, with gender differences being given limited consideration. This is in spite of accumulating evidence that there are a number of potential differences between women and men which may affect response to treatment, pharmacokinetics, toxicities and coping. There are also notable psychological, behavioural, social and structural factors that may have a unique impact on women living with HIV (WLWH). Despite our increasing knowledge of HIV and advances in treatment, there are significant gaps in the data relating specifically to women. One of the factors contributing to this situation is the under-representation of women in all aspects of HIV clinical research. Furthermore, there are clinical issues unique to women, including gynaecologic and breast diseases, menopause-related factors, contraception and other topics related to women's and sexual health.

**Methods:**

Using scoping review methodology, articles from the literature from 1980 to 2012 were identified using appropriate MeSH headings reflecting the clinical status of WLWH, particularly in the areas of clinical management, sexual health, emotional wellbeing and treatment access. Titles and abstracts were scanned to determine whether they were relevant to non-reproductive health in WLWH, and papers meeting inclusion criteria were reviewed.

**Results:**

This review summarizes our current knowledge of the clinical status of WLWH, particularly in the areas of clinical management, sexual health, emotional wellbeing and treatment access. It suggests that there are a number of gender differences in disease and treatment outcomes, and distinct women-specific issues, such as menopause and co-morbidities, that pose significant challenges to the care of WLWH.

**Conclusions:**

Based on a review of this evidence, outstanding questions and areas where further studies are required to determine gender differences in the efficacy and safety of treatment and other clinical and psychological issues specifically affecting WLWH have been identified. Well-controlled and adequately powered clinical studies are essential to help provide answers to these questions and to contribute to activities aimed at improving the health and wellbeing of WLWH.

## Introduction

It is often overlooked that more than 50% of adults living with HIV globally are women and, as women make up a higher proportion of new diagnoses than men, the number of infections among this group is increasing in many countries [1]. Regardless of the epidemiology, women have always been under-represented and even excluded in many areas of HIV clinical research. Numerous barriers have prevented women from being included in clinical trials of antiretroviral therapy (ART). Even when they are involved, the studies are often flawed [2], underpowered to provide gender analysis or simply not analyzed by gender to provide women-specific evidence. Where studies of women do exist, these are most often confined to the issue of pregnancy, a vital area of interest in relation to women, but not representing the totality of female experience. Overcoming these barriers is crucial to increase female participation in clinical research [3] and to provide gender-specific knowledge.

To critically assess the existing guidance and data relevant to women living with HIV (WLWH), the literature was reviewed to identify both the current consensus and areas for further study with regard to clinical management, sexual health, emotional wellbeing and treatment access as it relates to WLWH and research using a gender-based analysis. Issues around HIV and pregnancy were not included in this review. Based on the available evidence and current clinical practice, a social justice framework was used to propose a call to action to address key areas of direct concern to WLWH.

## Methods

This article was written using scoping review methodology [4] to provide a narrative account of available research in gender differences in HIV-related non-reproductive clinical factors, including ageing, emotional and sexual wellbeing, and other challenges faced by WLWH. A scoping review aims to achieve in-depth and broad results. During the process of identifying relevant studies and on-going literature review, search terms can be redefined to undertake more specific searches. The following five steps were used in scoping review methodology and repeated as necessary to ensure the literature was fully investigated: 1) identifying the research question; 2) identifying relevant studies; 3) study selection; 4) reviewing the data; and 5) collating, summarizing and reporting the results of the literature review.

In this study, MEDLINE was searched for article titles from 1980 to 2012. At the time of the review there were 331,546 papers mentioning the terms “HIV” or “AIDS.” Of these, only 112,474 mentioned the word “female” or “women,” whereas 120,896 mentioned the word “male” or “men.” The papers were divided into four sections – clinical management, emotional health, sexual health and treatment access – based on the use of diverse MeSH headings for each section, including “HIV”; “women” or “gender”; “antiretroviral response,” “antiretroviral toxicity,” “ageing,” “co-morbidities,” “sexual health,” “menopause,” “depression” or “emotional wellbeing”; and “treatment access.” Titles of papers were searched electronically to allocate potentially relevant articles for inclusion in a scoping review process [4]. The abstracts of the papers allocated to each section were scanned for relevance and subsequently a full review of the identified papers was conducted. The full review of papers was an iterative process in line with scoping review methodology [4] with articles being included based on relevance to the identified gap in the literature and author opinion of suitability for inclusion.

Relevant bibliographies, existing networks and HIV organizations, guidelines and abstracts were also reviewed from conferences, including Conference on Retroviruses and Opportunistic Infections (CROI), International AIDS Society Conference on HIV Pathogenesis, Treatment and Prevention (IAS), International AIDS Conference (IAC), Canadian Association for HIV Research (CAHR), European AIDS Clinical Society (EACS) and the International Workshop on HIV and Women from Adolescence through Menopause. Experts in the field were consulted regarding missing publications. Before the full articles were reviewed abstracts were scanned to determine whether they were relevant to the aims of the review and to eliminate those focusing on reproductive health, including preconception, contraception, maternal health and postpartum issues in WLWH. These reproductive health issues have been reviewed in detail elsewhere [5].

A social justice framework provides plans and strategies for how equal opportunities can be realized for vulnerable or under-represented population groups. This article provides a social justice framework for improving the care of WLWH. The non-reproductive challenges faced by WLWH and gaps in the literature are discussed so that researchers in this area can work towards improving the care of this population group. As this is a review article, the results and discussion are presented together. In addition, a summary of the gaps in the literature around the challenges faced by WLWH are included at the end of each of the sections on clinical management, sexual health, emotional health and treatment access.

## Inclusion of women in HIV clinical research

Women have been under-represented and excluded in many areas of HIV clinical research often to minimize the risk of harming the unborn child. However, clinical trials need to reflect the general population living with HIV and thus include an appropriate proportion of women, rather than simply extrapolating from predominantly male data [3, 6]. Although more and more pharmaceutical companies are conducting meta-analyses of specific ARTs by gender, and should be congratulated for doing so, many clinical studies involving women are still cohort or observational, rather than randomized, as well as being under-powered for gender comparisons [2]. While it is costly to conduct large trials to investigate gender differences, it is important that appropriately designed studies be conducted to help inform guidelines and treatment recommendations for these specific populations including women. This group faces social, historical, clinical, structural and practical barriers to their inclusion in HIV clinical research [3, 7]. Understanding these barriers and implementing strategies will help to establish equality of access and women's participation in research. Such strategies could include revising guidance on a woman becoming pregnant during a clinical trial to ensure that as much data as possible is analyzed prior to her withdrawal, and the provision of options for follow-up or even continuing in a trial if she remains on the same regimen. d'Arminio Monforte *et al*. [3] also advocates reviewing contraceptive requirements during a trial (so as to not place an unfair burden on the woman), having after-hours clinics and offering childcare provision (Table 1).

**Table 1:** Strategies to improve involvement of women in HIV clinical trials

Target group	Suggested strategy for increasing involvement of women
Clinical researchers	Ensure equal representation of men and women in earlier clinical studies (e.g. in Phase II) Women-only trials conducted at Phase III Protocols should be statistically powered to provide meaningful data on gender differences Protocols should be designed to remove unfair barriers to the involvement of women
Physicians	Work with clinical trial investigators to develop ways to improve appropriate recruitment of women into trials Put strategies into place to address barriers by catering to the specific needs of women (e.g. through providing childcare options)
Clinical trial sponsors	Collaborate with women investigators and centres responsible for the treatment of a high proportion of WLWH Provide specific tools to enable recruitment of more women
Regulatory authorities and ethical committees	Recommend that pharmaceutical companies, trial sponsors, and clinical trial investigators recognize and address the gender imbalance in HIV trials Recommend pharmaceutical companies and trial sponsors use female investigators
Journal editors	Encourage the reporting and publication of results for female participants in HIV clinical studies Incorporate a recommendation in the CONSORT guidelines* to include women in clinical trials of HIV treatment and to report female sub-analyses Ensure that publications represent and discuss women where possible and that the use of women-specific endpoints is investigated Encourage inclusion of a discussion of any evident differences between men and women that emerge from studies Include a women's health specialist on journal editorial boards Highlight where further research is required
Medical societies and congress organizers	Encourage the submission of study data on women in the congress call for abstracts Give priority to data on women when allocating scientific sessions and symposia Ensure scientific sessions and symposia address gender-specific issues related to treatment Ensure female investigators are part of congress organizing committees Give priority for oral presentation to high quality abstracts that address gender issues Monitor the numbers of female investigators and presenters attending congresses Monitor the proportion of women in studies that are reported at congresses
Advocacy groups	Raise awareness and highlight gaps in the reporting of data on women during scientific symposia Include articles in patient advocacy publications on the importance of the adequate representation of women in trials Provide peer education and support

* Available at: http://www.consort-statement.org/

Adapted from d'Arminio Monforte *et al*. [3] with permission.

Furthermore, there have been few women investigators involved as key participants in the pivotal ART trials. This is despite the fact that in many clinics female physicians look after a high proportion of female patients and efforts should be made to select a range of investigators. Gender issues in employment, promotion and glass ceiling effects may continually filter down into individual research areas and may need a conscious effort to rectify. This same phenomenon was seen in cardiac risk factor prevention research where over-inclusion of male subjects and of male investigators was only redressed after specific attention was drawn to the phenomenon, with resultant recruitment of a female only study arm [8, 9].

In addition to providing equal opportunities and the required support for the participation of women in HIV clinical studies it is important that the significance of reporting these data is recognized. A number of editorials have been published to encourage the representation of women and the investigation of women-specific endpoints where possible [7, 10, 11]. Furthermore, while journal articles are often limited by length, which may mean that gender-specific analyses are compromised, a number of journals do offer the opportunity for publication of supplementary material online, and this opportunity is often under-utilized.

## Gender differences and women-specific issues in HIV and its clinical management

Women and men differ in terms of susceptibility to HIV infection, the course of the infection, response to treatment, drug pharmacokinetics and toxicity [2, 12–14]. Poverty, immigration, isolation, socio-cultural norms and lack of education may also have a unique impact on WLWH and impact clinical outcomes [15, 16]. Clinical studies that define the impact of these factors will help inform therapeutic decisions in routine clinical practice.

### Virological and immunological response

**Figure 1:**
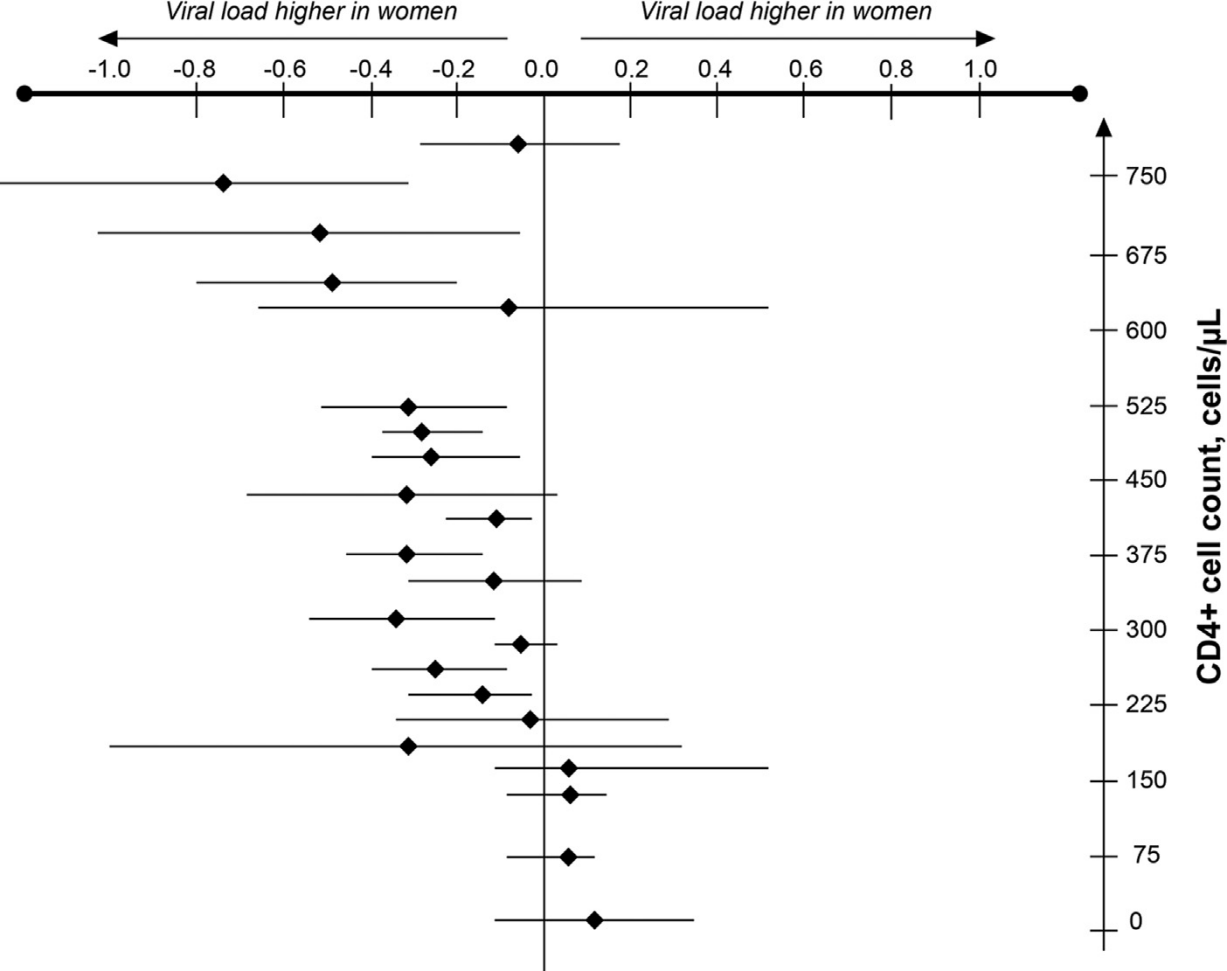
Meta-analysis of estimated differences in mean log_10_ HIV RNA levels between women and men [17].

**Figure 2:**
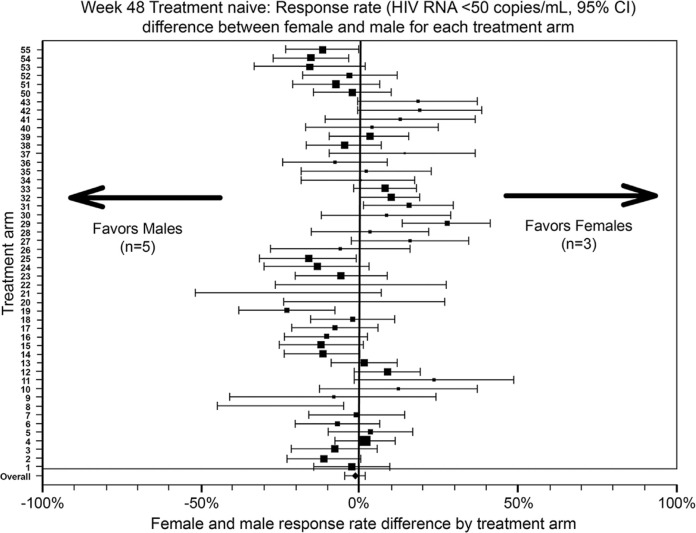
Gender-related efficacy differences in women and men [21]. The publisher for this copyrighted material is Mary Ann Liebert, Inc. publishers.

Gender differences exist in susceptibility to HIV infection as well as virological and immunological parameters [13]. A meta-analysis has concluded that women have lower baseline viral load than men, particularly early in the infection (Figure 1) [17]. Higher, lower and equivalent CD4 cell counts have been reported in various studies when women are compared with men [13, 18] and likely reflect confounding variables in addition to gender. These differences appear to have little bearing on the progression of the disease [13]. One cohort study showed that women had higher CD4 cell counts (*p*<0.001), lower viral loads (*p*<0.001) and a more favourable clinical profile (*p*<0.001) than men at baseline [19]. Women also had superior clinical (*p*<0.01), virological (*p*<0.01) and immunological (*p*<0.006) responses to ART in multivariate analyses after adjustment for other variables [19]. These gender differences could not be explained by factors such as adherence to therapy. In another cohort study, women were shown to survive longer and to experience a lower risk of both progression to AIDS and non-AIDS mortality following the introduction of highly active antiretroviral therapy (HAART) in 1996 [20]. These benefits occurred despite a slightly lower proportion of women on HAART compared with their male counterparts. A recent meta-analysis by Soon and colleagues (2012) [21], including 20,328 HIV-positive individuals from 40 randomized controlled trials, reported no overall difference in virological outcome at 48 weeks in those who were treatment naïve (Figure 2) or treatment experienced. Kwakwa and colleagues [22], however, conducted a meta-analysis of seven randomized controlled trials in treatment-naïve patients reporting virologic suppression or failure rates in both men and women and reported that women were 28% less likely to achieve virologic suppression compared to men (*p*<0.0001). In summary, given the inconclusive nature of the findings around the influence of gender on HIV disease progression further studies are required here.

### Drug pharmacokinetics and interactions

Women differ in their metabolic processes and renal and hepatic function compared with men [13, 14] and these differences may be accentuated by pregnancy, hormonal contraceptives, hormone replacement therapy (HRT) and ethnicity [23]. These factors should be considered when planning ART for women. Many current dosing guidelines and recommendations for ART initiation and monitoring highlight that women of reproductive age or those planning pregnancy may require different ART than men, however experience of adverse events potentially related to pharmacokinetics may also impact ART choice in women [24–26]. One would think that since gender differences exist in body size and composition, the differences in antiretroviral drug levels between women and men would be due to weight, however, it has been reported that there is no difference in pharmacokinetics according to body weight [27, 28] A number of studies suggest that females achieve higher plasma drug concentrations than males receiving the same doses, although this is not a consistent finding. A summary of these results are presented in Table 2. Further research is required in this area, including the interactions between ART and other therapies commonly used in women, such as HRT, antidepressants, statins, antidiabetic agents and chemotherapeutic medications.

**Table 2:** Gender differences in antiretroviral pharmacokinetic parameters

Treatment	Gender difference	Nature of difference in women versus men	Clinical implications
**Protease inhibitors**			
Ritonavir (SCG formulation)	Yes	31% ↑ median AUC_0–12 h_ (*p=*0.026) ↑ median C_max_ (*p=*0.032) 24% ↓ CL/F (*p=*0.026)	Gender differences in RTV concentrations might have a significant influence in settings where RTV is used to boost other protease inhibitors or in the magnitude and perhaps clinical significance of RTV drug-drug interactions [29]
Ritonavir (Tablet)	Yes	35% ↑ median AUC_0–12 h_ (*p=*0.012) ↑ median C_max_ (*p=*0.006) 26% ↓ CL/F (*p=*0.012)
Indinavir/r	Yes	30% ↓ clearance (*p*<0.05)	Initial dosage should be decided according to ritonavir intake and gender, prior to plasma concentration measurements [30]
Saquinavir/r	Yes	25% ↑ AUC_0–12 h_ (*p=*0.004) 3-fold ↑ C_min_ (*p*<0.001) ↑ C_max_ (*p*<0.05) ↑ Inhibitory quotient	A greater proportion of females than males had HIV RNA values ≤500 at week 16 of the study period [31–33]
Lopinavir/r	No	None	None [29]
Saquinavir	No	None	None [34]
Indinavir	No	None	None [35]
Atazanavir/r	Minimal	None	None [36]
Fosamprenavir	Minimal	None	None [37]
Fosamprenavir/r	Minimal	None	None [37]
**NRTIs and NNRTIs**			
Nevirapine	Yes	44% ↑ C_max_ (*p=*0.042) 1.5-fold ↑ ΔC (*p=*0.02) 0-22% ↑ random concentration 25% ↓ weight-adjusted clearance	Evidence suggests particular sensitivity of females to nevirapine toxicity, including rash and hepatotoxicity [38–40]
Efavirenz	Yes	↑ mean plasma concentration (*p*<0.001) 25% ↓ AUC_0–24h_ (*p=*0.05) Slight ↑ clearance (L/h)	Physicians should be aware of a higher risk for drug-induced toxicity in females [23]
Delavirdine	Yes	↓ mean intrinsic clearance (*p=*0.05)	None [41]
Etravirine	No	None	None [42]
Rilpivarine	No	None	None [26]
Lamivudine	Yes	1.6-fold ↑ in intracellular nucleoside triphosphates (*p=*0.002)	Physicians should be aware of a higher risk for drug-induced toxicity in females [43]
Zidovudine	Yes	2.3-fold ↑ in intracellular nucleoside triphosphates (*p=*0.002)
**Other agents**			
Enfuvirtide	Yes	35% ↓ clearance	Changes in exposure do not appear to affect efficacy or safety [41]
Maraviroc	ND	ND	ND
Raltegravir	No	None	None [44]
Elvitegravir/Cobisistat	ND	ND	ND

SGC=soft gel capsules; CL/F=apparent oral clearance; AUC=area under the curve; C_min_=minimum plasma concentration; C_max_=maximum plasma concentration; ΔC=difference between maximum and minimum concentrations (C_max_−C_min_); C_trough_=plasma concentration just before the next dose; ND=no data available.

### Antiretroviral drug toxicity

In general, women have been found to be more susceptible than men to developing ART-associated toxicities. Non-nucleoside reverse transcriptase inhibitors (NNRTIs) are related to an increased risk of rash and hepatotoxicity in women [13]. The thymidine analogues, DDI and DT4, are associated with lactic acidosis, especially in obese women, and have been reported to have fatal consequences in pregnant women when used in combination. This combination is no longer recommended in treatment guidelines and should be avoided in women, particularly pregnant women [45]. ART is also commonly associated with lipodystrophy and blood lipid abnormalities and ART-related toxicities have been found to occur at a higher frequency among women compared with men [46–51]. Table 3 provides an overview of gender differences in ART-associated toxicities. Further data in this area is required to help inform treatment decisions for women.

**Table 3:** Gender differences in antiretroviral toxicity

Regimen	Nature of difference in women versus men
**NRTIs and NNRTIs**	
NRTIs	↑ risk of developing body habitus changes (*p*<0.0001) [52]
Zidovudine (AZT), zalcitabine,	↑ likelihood of reducing dose or stopping didanosine-containing regimen [50]
didanosine	3-fold ↑ in risk for AEs because of didanosine (*p=*0.03) [45]
	↑ likelihood of developing anaemia with AZT (*p=*0.026) [53]
Nevirapine	7-fold ↑ in risk of rash (*p=*0.003) [54] 3 to 5 times ↑ likelihood of discontinuation due to rash (*p=*0.005) [54]
	2-fold ↑ in grade 4 elevations of liver enzymes [55]
Efavirenz	Potential risk of ↑ AEs due to ↑ plasma concentration [23]
	2.2 times ↑ risk of discontinuing treatment, in part due to psychiatric AEs [56]
**Protease inhibitors**	
Ritonavir	↑ frequency of AEs (*p=*0.008) [57]
Fosamprenavir (± ritonavir)	Minimal [37]
Darunavir/r	Nausea and vomiting more common (NS) [58] Trend of higher discontinuation rates due to AEs [58]
Atazanavir/r	Minimal [59]
Lopinavir/r	Minimal – slight ↑ in nausea and ↓ in diarrhea [59]
Protease inhibitor-containing ART	↑ mean TG (*p* <0.02) ↑ mean LDL (*p* <0.0001) ↑ mean Leptin (*p* <0.02) ↑ fasting insulin levels and LDL/HDL ratio (*p=*0.02) [49]
	1.5-fold ↑ in risk of development of lipodystrophy syndrome [47]

NRTIs=nucleoside reverse transcriptase inhibitor; NNRTI=non-nucleoside reverse transcriptase inhibitor; ART=antiretroviral therapy; AE=adverse event; TG=triglycerides; LDL=low-density lipoproteins; HDL=high-density lipoprotein, NS=non-significant.

### Other gender differences in HIV

#### HIV clades

Globally, most WLWH are from resource-poor countries where non-B HIV clades are more common, with implications for the mode of HIV transmission. For example, clades A, C, D and E are transmitted primarily via heterosexual sex in Asia and Africa [60]. Inter-clade genetic variation may be an important factor in the development of drug resistance [60]. Most studies of ART have been conducted in patients with clade B virus and future studies should include an appropriate distribution of viral subtypes. Studies in pregnant and non-pregnant WLWH are needed to confirm if drug resistance is clade-specific and also to determine if there are any gender differences.

#### Immune activation

Immune activation is strongly associated with disease progression in HIV [61]. Gender differences in the activity of Toll-like receptors may account for higher immune activation in women compared with men at a given HIV-1 viral load. The higher level of T-cell activation may provide a mechanism by which the same level of viral replication might result in faster HIV-1 disease progression in women compared with men [62].

#### Tropism

HIV infection of macrophages and T-helper lymphocytes is essential to HIV pathogenesis. The viral tropism has been shown to affect disease progression rate but no association between HIV tropism and gender has been reported [63]. An HIV co-receptor switch can occur during pregnancy in a manner indistinguishable from that seen in non-pregnant patients [64].

#### Viral reservoirs

Several HIV reservoirs exist in the body, for example, blood plasma and the genital tract, where the virus may continue to replicate even in those receiving ART. It appears that there can be drug-resistant mutations and non-drug resistant mutations in the female genital tract which are not present in the plasma [65]. Thus treatments that can achieve therapeutic concentrations within this compartment may have important implications for disease transmission and controlling local viral replication [66]. Development of advanced techniques for measuring viral load in the female genital tract means that more data are becoming available in this area. A recent review has summarized differences in ART deposition and highlights that male and female data are inconsistent and further research is required to elucidate the differences [66].

### Women, HIV, ageing, co-morbidities and menopause

In Western Europe, the proportion of women aged ≥50 years accounting for new HIV cases rose from approximately 6 to 9% between 2002 and 2006 [46]. Consequently, HIV is now managed alongside the typical co-morbidities of ageing, for example, renal, metabolic and cardiovascular disease; osteoporosis; and neurocognitive changes. Co-morbidities are more common among those with HIV than in the general population. The prevalence of co-morbidities in those with HIV is similar to that observed among persons in the general population who are 10 years older [67]. HIV-specific co-factors (lower nadir CD4 cell count and more prolonged ART exposure) have been identified as risk factors [68]. Hepatitis C virus co-infection is a major source of morbidity and mortality in people with HIV, since it accelerates the progression to symptomatic liver disease and cirrhosis, as well as impacting the use of ART [69]. However, we know little about gender differences. The data support the need for earlier screening for non-AIDS-related co-morbidities in those with HIV. Furthermore as these conditions require their own treatments, an awareness of potential drug-drug interactions is also important to ensure that care is appropriate for each individual person. There have been limited studies to date assessing such co-morbidities in women or their gender differences in HIV.

WLWH may have an increased risk of many forms of cancer compared with men, classified as AIDS-defining (those indicative of severe immunosuppression) and non-AIDS defining [70]. The former category includes Kaposi's sarcoma, lymphomas and invasive cervical cancer [29]; the prevalences of which are relatively high among WLWH [71]. Although HIV infection directly and indirectly affects breast tissue, the incidence of breast cancer appears not to be increased in WLWH. Indeed, several large studies have reported a slightly lower incidence of breast cancer compared to the general population [72–74]. The age at breast cancer diagnosis is similar in WLWH and the general population, after age adjustment for at-risk populations [75], and screening for breast cancer should follow standard, age-appropriate screening recommendations that apply to the general population. However, the interaction between ART and chemotherapy needs to be assessed further. Also, further research in this area will determine whether better supportive care is required for this population in the future. WLWH are more likely to have human papillomavirus (HPV) than HIV-negative women and the incidence of the cellular changes that precede cervical cancer are 4 to 5 times higher among WLWH than HIV-negative women [76–78]. Notably, the incidence of cervical cancer has not decreased since the introduction of ART, highlighting the importance of regular screening [76, 79]. Furthermore, some strains of HPV are associated with anal and oropharyngeal cancers in WLWH, [80] necessitating anal pap smears. Evidence suggests that HPV-related anal cancer will not decline among those living with HIV and anal pap smears will help identify cases [76]. The effectiveness of HPV vaccination in WLWH is currently under investigation. It is thought that, as most women coinfected with HIV/HPV do not have all the HPV types included in the vaccine, they are likely to receive some benefit [69, 81].

In earlier studies, neurocognitive impairment is more common among WLWH compared with their male counterparts [82] and women are reported to have a more rapid progression of neurologic signs and symptoms compared with men [83]. A recent study in Zambia [84] showed a neuropsychological deficit effect for HIV-positive versus HIV-negative groups and noted that it was only the female seropositive participants who showed this HIV effect. Not taking ART, increasing age and depressive symptoms were associated with an increased risk of neuropsychological impairment [85]. Neurocognitive impairment and depression can lead to reduced ART adherence which, in turn, further impairs neurocognitive health [86]. Consideration of these factors in the care of WLWH is important. Some studies show that dual infection with HIV and Hepatitis C may affect cognition [87], yet the largest women only study (*n =*1338) recently found no such effect [88] showing the importance of on-going evidence gathering and specific data on women.

As HIV mortality rates have decreased since the introduction of ART, more WLWH are reaching menopause. A summary of the key literature on HIV and menopause is provided in Table 4. Studies report that WLWH may experience menopause at an earlier age [89–91], with a greater degree of symptoms and with a different reproductive hormone profile than HIV-negative women [92–94]. Some menopausal characteristics are similar to symptoms of HIV infection or to side effects of HIV medication such as menstrual cycle irregularities, skin and hair changes, emotional changes or night sweats. Menopausal WLWH have multiple, potentially additive factors that predispose them to metabolic complications, including osteoporosis, and lipid and glucose disturbances. These factors include the consequences of HIV itself, impact of ART, loss of the protective effects of estrogen which are found in menopausal women and adverse effects of HRT. Studies are needed to confirm whether these factors are clinically significant, and whether they do indeed translate into increased bone fractures and cardiovascular events. In addition, clinicians require a clear understanding of the possible interactions between HIV and hormones and how these influence disease progression and treatment [12].

**Table 4:** Summary of key menopause studies in WLWH

Objective	Study Population	Findings
**Natural history**		
To study prevalence and factors associated with early menopause in women from the DIDI Study [30]	352 HIV+ women aged≤46 years	The prevalence of early menopause (7.7%) was comparable with that reported in the Italian general population (7.1%); a higher proportion of menopause was observed in women≤40 years (5.2% vs. 1.8%) Advanced stage of HIV was the main predictor of early menopause
To describe the characteristics of postmenopausal WLWH and to investigate the factors associated with an earlier onset of menopause [89]	404 HIV+ women (69 naturally postmenopausal at time of study)	Earlier onset of menopause was associated with IDU, ethnicity and CD4 cell count <200 cells/mm
To characterize prolonged amenorrhea from ovarian failure and other causes and to estimate if HIV serostatus is a risk factor for amenorrhea in HIV+ and HIV− women [90]	1431 women (1139 HIV+ and 292 HIV−)	HIV infection is associated with amenorrhea in WLWH and may be positively associated with ovarian failure in women with amenorrhea
To study the relationship of HIV infection with the onset of natural menopause [91]	571 (53% HIV+)	HIV infection and immunosuppression were associated with an earlier age at onset of menopause (46 years vs. 47 years)
To obtain information on the prevalence of anovulation and early menopause and on pituitary-gonadal function among WLWH [95]	Stored serum samples from 52 WLWH aged 20 to 42 years who participated in selected ACTG protocols	Study demonstrated a relatively high frequency of anovulation in WLWH with trends suggesting a relationship between lower CD4 T-cell counts and anovulation 8% of the subjects had presumed early menopause – compatible with the frequency in the general population
To examine the median age of menopause, factors associated with postmenopausal status, and the prevalence of menopausal symptoms in WLWH [64]	120 HIV+ women aged 40 to 57 years	Median age of menopause was 50 years 80% prevalence of hot flashes observed in WLWH compared to 38–69% in the general population
**Menopausal symptoms**		
To assess the effects of HIV infection and ART on change in BMD in postmenopausal women [92]	Prospective cohort study of 128 (73 HIV+, 55 HIV−) postmenopausal Hispanic and African-American women	HIV+ postmenopausal women had lower BMD, increased bone turnover, and higher rates of bone loss than HIV− women
To examine the association of HIV infection, drug use, and psychosocial stressors with type and frequency of menopausal symptoms [93]	536 women not on hormone therapy (54% HIV+)	WLWH reported more menopause symptoms than HIV− women; symptoms were less frequent in women with more advanced HIV disease Depressive symptoms and negative life events were also independently associated with symptoms
To evaluate the prevalence and factors associated with menopause symptoms in WLWH [94]	251 women (96 HIV+; 155 HIV−) aged=40 years	Menopausal symptoms were common in WLWH, particularly psychological and vasomotor symptoms HIV infection was independently associated with menopause symptoms
**Treatment response**		
To study initial treatment responses to ART in postmenopausal WLWH [96]	267 WLWH (220 pre-menopausal and 47 post-menopausal)	No significant difference in changes in CD4 cell counts or HIV type 1 RNA levels between groups

ACTG=Adult AIDS Clinical Trials Group; IDU=intravenous drug user; BMD=bone mineral density.

## Summary of knowledge gaps and research needs related to gender differences and women-specific issues in HIV and its clinical management

- Additional clinical trials of HIV therapies which incorporate endpoints to investigate gender differences, such as:
  - Immunological, virological and clinical response to ART
  - Effects of different ARTs on various body systems
- Reporting of HIV research findings disaggregated by gender to identify differences and impact in women, either in the main body of the paper or as an online supplement
- Impact of gender differences on routine clinical management
- Further meta-analyses of multiple, drug specific trials to better assess potential differences in response, adherence, side effects and long-term toxicity
- How different ARTs interact with
  - Endogenous and exogenous sex hormones
  - Other drugs commonly used in women
- Management of co-morbidities and diseases of ageing in WLWH
- Definitive research on the natural history and management of menopause for WLWH

## Sexual health in WLWH

The World Health Organization defines sexual health as: “a state of physical, mental, and social wellbeing in relation to sexuality. It requires a positive and respectful approach to sexuality and sexual relationships, as well as the possibility of having pleasurable and safe sexual experiences; free of coercion, discrimination, and violence” [97]. Sexual health in WLWH encompasses a broad range of topics [98], including the impact of HIV on sexual desire or satisfaction, a negative association of sex with HIV, feelings of guilt and shame, resentment towards a sexual partner, and infertility. However, research in this area is limited and what has been conducted has tended to focus on the more physical issues, such as the effect of HIV infection on the natural history of reproductive illnesses. The psychosocial issues of women's sexual health, such as the impact of HIV on sexual desire and satisfaction, have received relatively little attention [55, 99–102]. From the available data, it appears that WLWH experience significantly lower sexual satisfaction than do their HIV negative counterparts [100]. Furthermore, researchers report that sexual desires change over time, with patients reporting diminished sexual desire in the early stages of ART treatment compared with later on in their treatment, when their health has improved [55]. Reasons for this reduced desire include misconceptions and fears of the consequences of engaging in sex on health, fear of transmitting the virus to a HIV-negative partner, insufficient energy, fear of superinfection, lack of trust in partners, feelings of unattractiveness and no longer considering sex an important part of life [55, 101]. Sexual health is also related to the decision to disclose and the impact of this on relationships, as well as the potential fear of stigma and discrimination as well as criminalization. Different jurisdictions have different laws related to the criminalization of HIV non-disclosure and transmission. Many criminal laws are broadly written such that they may enable criminal prosecution of vertical transmission [103]. Such laws may in fact harm women rather than assist them and have a negative impact on public health and human rights [104, 105]. Reproductive health for WLWH is another important topic that we have reviewed in a separate publication [106].

## Summary of knowledge gaps and research needs related to sexual health for WLWH

- Research efforts should focus on the sexual health of WLWH to support healthy sexuality in this population
- Significant work is required in the area of HIV disclosure and criminalization and how it is related to healthy sexuality for WLWH

## Emotional health in WLWH

The term emotional health encompasses a state of emotional and psychological wellbeing in which an individual is able to use his or her cognitive and emotional capabilities to function in society and meet the normal demands of everyday life. All people with HIV face numerous emotional health challenges, which include, but are not limited to, coming to terms with their diagnosis, the impact of the disease on family and friends, and fear of disclosure. These issues can also have a negative impact on treatment-seeking behaviour, quality of life (QoL), adherence to medication and clinical outcomes.

Emotional health challenges range from mild-to-moderate depression, post-traumatic stress disorder [107, 108] and anxiety [109] to severe mood and psychotic disorders, as well as suicidal challenges [110]. The rates of such emotional challenges are elevated in the presence of HIV, with recent reviews reporting prevalence rates for depression between 0 and 80%, depending on the diagnosis tool used [108]. In addition, a large epidemiological study (*n=*2890; 38.5% women) reported that the prevalence of depression in those with HIV was nearly double what has previously been reported in the general population in Europe when using similar screening tools (approximately 8.6%) [19]. Trauma and posttraumatic stress disorders are frequent in WLWH, and may even predict poor HIV-related health outcomes and sexual behaviours with a high transmission risk [111]. Unfortunately, we know little of the gender differences in these issues. However, WLWH appear to be susceptible to depressive symptoms and depression is more prevalent in this group compared with HIV-positive men [25]. When compared to men, women show higher levels of global distress and psychological adjustment challenges [112]. In addition, the rate of stigma and discrimination reported among WLWH is higher than men [5]. This can have a strong impact across several areas of a woman's life, including health and wellbeing, and psychological and social effects [113]. Stigma may also then create a barrier to access supportive and adaptive pathways such as reliable relationships or supportive social groups. Furthermore, a high prevalence of domestic violence against women with HIV, which can have an impact on emotional wellbeing, has been reported. One study observed that the estimated rate of intimate partner violence among women was 55.3%, which is more than twice the US national rate [111].

Early assessment of psychiatric symptoms and appropriate reaction and treatment provision in people living with HIV might improve overall wellbeing. High levels of psychological distress, low health-related QoL, unwillingness to seek help and financial support, sexual problems and childcare issues have been reported among women [114, 115]. Counselling and cognitive-behavioural interventions can reduce stress, increase overall QoL [1, 25, 116–118] and promote positive emotional health and wellbeing as well as reducing HIV risk behaviours. Peer support and mentoring are useful when cultural and social barriers preclude professional counselling.

Some ART regimens are associated with a higher prevalence of depressive symptoms [25] with implications for treatment adherence and ultimately clinical outcomes [119]. Compared with men living with HIV and suffering from depression or experiencing stigma, women are less adherent to drug therapy, which may be improved with psychiatric care and antidepressant therapy [113, 120]. In fact, HIV-related stigma and discrimination is thought to have a negative impact on emotional, psychological and social wellbeing [113] and women with HIV are shown to experience gender-based stigma [121]. This may be linked to sub-standard treatment and can also present a barrier to accessing and retaining healthcare services and social supports [122–124] for those living with HIV. The authors conclude that reducing HIV-related stigma is therefore key to promoting the health of people with HIV and further research is required in this area to determine why women may experience higher rates of stigma [5].

Healthcare professionals need to recognize and provide appropriate and individualized intervention to maintain emotional wellbeing and help improve overall outcomes in WLWH.

Positive emotional health and wellbeing among WLWH enhances the ability to cope with a diagnosis of HIV, to adjust their lifestyle to suit treatment, and to improve resilience to any stigma and discrimination. A range of interventions, both pharmacological and behavioural, may be required to help WLWH cope with their lives and plan for the future.

## Summary of knowledge gaps and research needs related to emotional health for WLWH

- Assessment of emotional wellbeing of WLWH and strategies for the optimal management of emotional health problems in WLWH alongside ART
- Programmes that address violence against women
- Improved understanding of the psychosocial factors that influence medication adherence in women
- Innovative approaches to addressing the stigma and discrimination faced by WLWH

## Treatment access

In Europe, Canada and the United States, up to 30, 27 and 20%, respectively, of HIV cases remain undiagnosed [43, 95]. Late diagnosis may cause a 10-fold increase in mortality and makes it more likely that the virus will be transmitted to others [57]. For women, the entry points for testing, diagnosis and treatment of HIV are regular health clinics and during antenatal care. Commonly, women seek routine healthcare for contraception, cervical cancer screening, pregnancy, menopause, fertility treatment and sexually transmitted infections. They may also seek help for depression and anxiety. These points of entry into the healthcare system make good opportunities to engage women in testing and care. Many of these facilities however do not routinely offer or recommend HIV testing, representing serious missed opportunities. However, healthcare inequalities are common, and women who are homeless, migrant or from ethnic minorities, generally have poor access to healthcare. Improvements are also needed in the access women have to the appropriate gender-specific information enabling them to make informed care-related choices [118, 125].

Factors such as community perception; religious beliefs; financial issues; language; competing health, family and childcare commitments; and continuity of care can prevent or motivate women in seeking access to HIV treatment. These need to be considered since they may affect the survival and QoL of those living with HIV. In the United States, high levels of stigma are associated with poor access to care and low medication adherence [5]. Race and ethnicity are also important as people from racial and ethnic minorities lose 1.5 years more of life than Caucasians due to late initiation and premature discontinuation of HIV therapy. These racial and ethnic disparities are even more pronounced among women [76]. Furthermore, the transition from paediatric to adult care services has implications for a young woman's psychosocial and educational needs [126]. However, little information on transitioning paediatric HIV patients is available.

### Treatment access in the developing and developed world

Of those requiring access to treatment only 31% of those in low- and middle-income countries were on treatment by 2007 [124]. As of 2009 there was still a discrepancy between the developed and developing world, with only 42% of people having access to treatment in the latter [124]. However, in some areas, great improvements in treatment access have been made with 2 million life years saved in sub-Saharan Africa between 2002 and 2008 [124].

Supply chain issues [117], insufficient finances and lack of healthcare infrastructures for testing, treatment, appropriate patient education and training of healthcare professionals are all barriers to universal treatment access. Women, in particular, are often less able to travel and are more economically vulnerable than men, compounding the above issues. Achieving global equality of access to HIV treatment requires investment and commitment from all stakeholders.

## Summary of knowledge gaps and research needs related to treatment access for WLWH

- Develop effective and supportive policy environments to address the specific needs of women to support their HIV testing and care
- Ensure healthcare systems have the structure and tools to enable enhanced responsiveness to the specific needs of WLWH
- Develop programmes aimed at overcoming obstacles and allowing equitable and improved access to care for women and that offer services addressing women's needs

## Conclusions

The impact of gender on HIV undoubtedly requires further research. Women and men potentially differ in the course of their HIV infection, their response to treatment and drug pharmacokinetics, all of which are compounded by social and behavioural factors. Data on the effects of ART on women are limited and require continued evaluation. Furthermore, an enhanced understanding of what this group needs in terms of sexual health issues and the management of co-morbidities associated with ageing is also required. Regarding psychological care, the emotional wellbeing of women can be affected by issues of stigma, discrimination, violence, self-resilience and post-traumatic stress. These factors can impair QoL and potentially HIV drug adherence, ultimately affecting clinical outcomes; therefore more research is required. Furthermore, all WLWH are entitled to equitable access to HIV, sexual, reproductive and general healthcare. To achieve this we have to identify what changes are required in healthcare policy and the tools and programmes necessary to implement these changes. The answers to these important questions will help inform the necessary changes to the treatment guidelines for the effective care of WLWH.
